# Biomaterial-Based Regulation of Mesenchymal Stromal Cell Hepatic Differentiation for Liver Regeneration

**DOI:** 10.3390/bioengineering13101157

**Published:** 2026-10-03

**Authors:** Lexi Balster, Ramy A. Hinna, Ayman A. Hassan, Gamal Shiha, Ahmed Lotfy

**Affiliations:** 1 Department of Biology, Hal Marcus College of Science and Engineering, University of West Florida, Pensacola, FL 32514, USA; ldb47@students.uwf.edu (L.B.); rah96@students.uwf.edu (R.A.H.); 2 Egyptian Liver Research Institute and Hospital (ELRIAH), Mansoura 35661, Egyptg_shiha@hotmail.com (G.S.); 3 Higher Technological Institute of Applied Health Sciences, Mansoura 35661, Egypt; 4 Internal Medicine Department, Faculty of Medicine, Mansoura University, Mansoura 35516, Egypt

**Keywords:** mesenchymal stromal cells, hepatic differentiation, hepatocyte-like cells, biomaterials, extracellular matrix, liver regeneration, tissue engineering

## Abstract

Liver failure and advanced chronic liver disease remain major causes of morbidity and mortality, while donor organ shortages, procedural complexity, cost, and the need for lifelong management after transplantation limit the availability of liver transplantation. Mesenchymal stromal cells (MSCs) have attracted substantial interest as a cell source for liver regeneration because of their accessibility, expansion capacity, immunomodulatory properties, and regenerative secretome. MSC-based hepatic differentiation, however, remains challenging, and cells generated in vitro frequently display an incomplete hepatocyte phenotype and limited maturation. Increasing evidence indicates that soluble differentiation factors alone are insufficient to reproduce the complex microenvironmental cues that regulate hepatic cell fate. Biomaterials can provide three-dimensional architecture, extracellular matrix (ECM) ligands, mechanical cues, cell-adhesive sites, and controlled presentation of growth factors, thereby acting as more than passive carriers of cells. In this mini-review, we summarize studies investigating natural and synthetic biomaterials for MSC hepatic differentiation, with emphasis on collagen-, gelatin-, heparin-, alginate-, and decellularized ECM-based systems as well as synthetic polymeric scaffolds such as poly(lactic-co-glycolic acid) and poly(L-lactic acid)-co-poly(ε-caprolactone). We discuss how biomaterial composition and three-dimensional organization may cooperate with hepatic differentiation media to promote hepatic phenotype and function. We also highlight an important distinction between expression of hepatic markers and acquisition of mature hepatocyte function, and identify opportunities for biomaterial systems that integrate biochemical, biophysical, and paracrine cues to achieve more reproducible hepatic maturation and clinically relevant liver regeneration.

## 1. Introduction

The liver performs diverse metabolic and synthetic functions, including the regulation of lipid and carbohydrate metabolism, plasma protein synthesis, xenobiotic metabolism, detoxification, and bile production. Although the liver possesses a remarkable regenerative capacity, persistent or severe injury can overwhelm this capacity and ultimately lead to fibrosis, cirrhosis, and liver failure [1,2]. Viral hepatitis, alcohol-associated liver disease, metabolic disorders, medications, and autoimmune injury are among the major causes of acute and chronic hepatic injury. For patients with advanced liver failure, transplantation remains an established treatment, but its broader application is constrained by donor-organ scarcity and other clinical and socioeconomic limitations. These challenges have stimulated the development of cell-based and tissue-engineering strategies for liver regeneration [2,3].

Stem-cell-based approaches have been explored as alternatives or complements to conventional transplantation. Stem cells are broadly characterized by self-renewal and multilineage differentiation potential, although their developmental potential varies substantially among stem-cell populations [4,5,6]. Adult stromal populations, particularly mesenchymal stromal cells (MSCs), have attracted considerable attention because they can be isolated from multiple tissues, expanded ex vivo, and investigated in allogeneic settings. In accordance with current International Society for Cell & Gene Therapy (ISCT) nomenclature, the term “mesenchymal stromal cells” is used here rather than “mesenchymal stem cells,” because designation as stem cells requires more rigorous demonstration of stemness [7]. MSCs also possess important immunomodulatory and paracrine properties, and their secretome may contribute substantially to tissue repair independently of direct differentiation [8].

MSCs derived from bone marrow and other tissues have been investigated for liver repair, and preclinical and clinical studies have reported beneficial effects in models and patients with liver injury [9,10]. Nevertheless, a major limitation of MSC hepatic differentiation is the incomplete acquisition of a mature hepatocyte phenotype. Cells generated by conventional induction protocols may express hepatic markers without fully reproducing the metabolic, polarization, transport, and cytochrome P450 functions of primary hepatocytes. Thus, improving the microenvironment in which MSCs undergo hepatic differentiation is an important objective for regenerative medicine.

The extracellular matrix (ECM) is a central component of this microenvironment. In addition to providing structural support, the ECM regulates cell adhesion, survival, migration, proliferation, and differentiation through biochemical and mechanical signals. Liver ECM composition changes during development, homeostasis, injury, and regeneration, providing a biological blueprint for engineering hepatic microenvironments [11,12,13]. Biomaterials can reproduce selected features of this environment while providing controllable architecture, stiffness, porosity, degradation, and spatial organization. Three-dimensional organization may also enhance cell–cell interactions and hepatosphere formation, which can improve cell survival and hepatic function [14]. Biomaterial-based delivery may further improve local cell retention and facilitate placement of cells within or adjacent to injured liver tissue [15,16].

**Figure 1:**
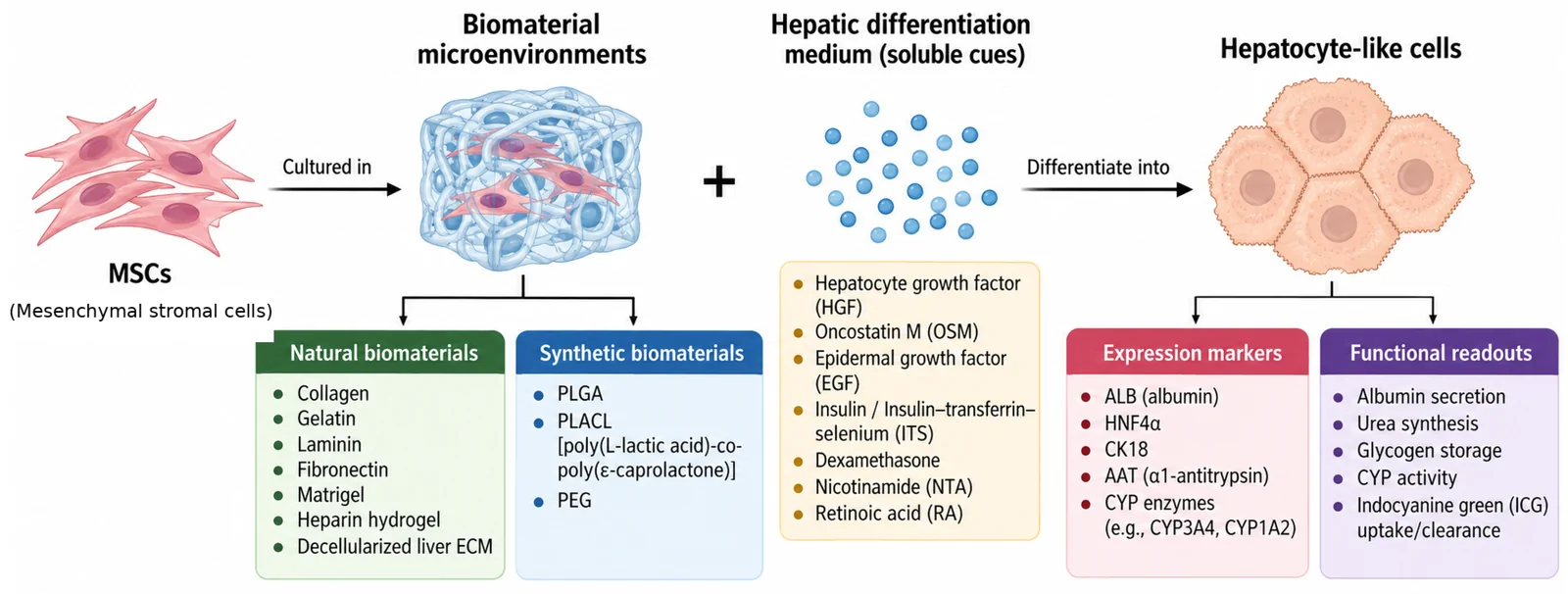
Conceptual framework for biomaterial-guided MSC hepatic differentiation and functional assessment. Biomaterial microenvironments integrate natural or synthetic material properties with soluble hepatogenic cues to regulate MSC adhesion, organization, signaling, and hepatic differentiation. The framework distinguishes hepatic identity from function: hepatocyte-like cells should be characterized using multiple expression markers (e.g., HNF4α, ALB, AAT, CK18, and CYP enzymes) together with quantitative functional readouts such as albumin secretion, urea synthesis, glycogen storage, CYP activity, and indocyanine green uptake/clearance. These complementary endpoints help distinguish acquisition of a hepatic-like phenotype from the development of hepatic function and maturation.

Accordingly, this mini-review focuses on the role of biomaterials in guiding MSC hepatic differentiation and the generation of hepatocyte-like cells. We retain the useful distinction between natural and synthetic biomaterials and emphasize how material composition and three-dimensional architecture can complement soluble hepatic differentiation cues. We also identify key limitations in the current literature and opportunities for designing more instructive biomaterial microenvironments for hepatic maturation (Figure 1).

## 2. MSCs as a Platform for Hepatic Differentiation and Liver Regeneration

MSC hepatic differentiation has been investigated both in vitro and in vivo using combinations of growth factors, cytokines, and chemical compounds. Reported hepatic-inducing factors include hepatocyte growth factor (HGF), fibroblast growth factors (FGFs), epidermal growth factor (EGF), oncostatin M (OSM), leukemia inhibitory factor, and insulin-like growth factor (IGF) [17]. In vivo studies have reported hepatic-marker expression following administration of human umbilical cord-derived MSCs to models of liver fibrosis, supporting the ability of MSCs to acquire features of the hepatic lineage [18]. Experimental work has also demonstrated hepatic differentiation of human MSCs within injured liver environments [19,20].

Importantly, evidence for MSC-to-hepatocyte conversion should be interpreted cautiously. Detection of hepatic markers after transplantation does not by itself establish the generation of fully mature hepatocytes, and experimental systems must distinguish direct differentiation from paracrine stimulation, cell fusion, or other forms of host-tissue remodeling. The literature therefore supports the concept that MSCs can acquire hepatocyte-like characteristics, but the extent to which they become functionally equivalent to primary hepatocytes remains an important question.

A related conceptual limitation is the developmental distance between MSCs and hepatocytes. MSCs are conventionally associated with mesodermal differentiation, whereas hepatocytes arise from definitive endoderm during embryonic development [21]. Consequently, differentiation of MSCs into hepatocyte-like cells represents a cross-lineage transition rather than conventional differentiation within established mesenchymal lineages. Although numerous studies have reported acquisition of hepatic morphology, markers, and selected functions by MSCs under hepatogenic conditions, the extent to which these cells acquire a stable and fully mature hepatocyte phenotype remains debated. Expression of a limited number of hepatic markers alone is insufficient to establish complete hepatic conversion, and, particularly in in vivo studies, alternative mechanisms such as cell fusion should be considered. Therefore, claims of MSC-to-hepatocyte differentiation are strengthened by demonstrating multiple hepatic lineage markers together with quantitative liver-specific functions and comparison with mature hepatocytes [19,22,23,24]. Accordingly, improvements in hepatic markers or selected functions within biomaterial-based systems should be interpreted as evidence of enhanced hepatic differentiation toward a hepatocyte-like phenotype rather than, by themselves, proof of complete lineage conversion or functional hepatocyte replacement in vivo.

The local microenvironment appears to be particularly important. MSC hepatic differentiation is influenced not only by soluble factors but also by ECM composition, cell–cell interactions, and three-dimensional organization [19,20,25,26]. This observation provides the rationale for using biomaterials as instructive microenvironments rather than simply as passive carriers. An effective scaffold can potentially coordinate soluble hepatic signals with ECM ligands and biophysical cues, thereby improving both differentiation efficiency and functional maturation.

## 3. MSC Sources Used in Biomaterial-Based Hepatic Differentiation

MSCs used in hepatic tissue-engineering studies have been obtained from several sources, including bone marrow, adipose tissue, umbilical cord, and Wharton’s jelly. Bone marrow was among the earliest and most extensively investigated sources, and BM-MSCs have demonstrated multilineage differentiation capacity and potential utility in regenerative medicine [27,28]. Adipose-derived MSCs offer the practical advantage of relatively abundant tissue availability and minimally invasive harvesting [29]. Umbilical cord- and Wharton’s jelly-derived MSCs represent additional sources that may be attractive because of their accessibility and proliferative properties. Beyond hepatic differentiation potential, tissue origin can influence properties that are directly relevant to manufacturing and therapeutic performance. Comparative studies have identified source-dependent differences in expansion kinetics and lentiviral transduction performance between bone marrow- and adipose-derived MSCs [30], while broader comparisons have demonstrated source-associated differences in immunomodulatory activity, gene expression, and secretome composition [31]. Thus, MSC source should be treated as a biological design variable rather than a simple interchangeable input, and source-specific attributes should be reported when biomaterial platforms are compared.

The available biomaterial studies illustrate that no single MSC source has emerged as universally optimal for hepatic tissue engineering. BM-MSCs have been evaluated in alginate, collagen-containing polymeric scaffolds, and decellularized matrices [32,33,34,35], whereas adipose-derived MSCs have been studied in liver-derived ECM, gelatin/laminin cryogels, heparin-based hydrogels, and ECM-functionalized synthetic hydrogels [29,36,37,38,39]. UC-MSCs and WJ-MSCs have also been investigated in three-dimensional alginate and heparin/collagen systems [40,41]. Differences in donor age, tissue source, culture history, and baseline phenotype may contribute to variability among studies and should be considered when comparing biomaterial platforms.

## 4. Soluble Hepatic Cues as Partners of Biomaterial Signaling

Hepatic differentiation protocols generally combine soluble factors with a defined culture environment. HGF, FGF, EGF, OSM, insulin/insulin-transferrin-selenium (ITS), dexamethasone, nicotinamide, and other compounds have been incorporated into one-step or sequential induction protocols [42,43,44,45,46,47,48]. Sequential protocols are particularly relevant to biomaterial design because they attempt to reproduce the changing signals encountered during lineage specification and maturation.

The precise contribution of each factor depends on the differentiation stage and MSC source. EGF and FGF family members can support early proliferation and lineage specification, whereas HGF and OSM are commonly associated with hepatic differentiation and maturation. Dexamethasone and ITS are frequently incorporated during later stages to support acquisition or maintenance of hepatic characteristics [42,43,44,45,46,47,48]. Rather than viewing differentiation media and biomaterials as independent variables, biomaterial systems should be considered as platforms that can modify the spatial and temporal presentation of these soluble cues. This interaction is particularly important for heparin-containing materials, which can bind and retain growth factors, and for three-dimensional scaffolds that establish local concentration gradients and cell–cell interactions.

## 5. Biomaterials for MSC Hepatic Differentiation

### 5.1. Natural Biomaterials

Natural biomaterials are attractive for hepatic tissue engineering because they can provide biochemical signals resembling those found in native ECM. The liver ECM contains collagenous and non-collagenous proteins, including laminins and fibronectin, as well as proteoglycans and glycosaminoglycans [11]. Importantly, ECM composition and organization change during development and liver regeneration, indicating that ECM signals participate actively in tissue-specific gene regulation and repair [12,13]. Natural biomaterials can therefore provide not only structural support but also ligands that influence adhesion, survival, cytoskeletal organization, and differentiation.

Collagen is one of the most widely used ECM-derived materials. Three-dimensional collagen environments can support cell organization and maintenance of liver-specific functions [49]. Collagen coatings can also enhance MSC adhesion to culture surfaces, further supporting their use as bioactive interfaces for cell attachment and biomaterial functionalization [50]. In MSC hepatic differentiation, collagen has also been incorporated into synthetic scaffolds to combine biological recognition with controllable polymer architecture. BM-MSCs cultured on collagen-coated PLGA scaffolds acquired hepatic markers, including ALB, AFP, CK18, CK19, G6PD, HNF4, and cytochrome P450-associated markers [34]. This study illustrates an important principle of biomaterial design: a synthetic scaffold can become more biologically instructive when functionalized with an ECM protein.

Gelatin, a denatured derivative of collagen, provides a biodegradable and readily processable alternative while retaining collagen-derived biochemical motifs [51]. Gelatin cryogels have been used to create porous three-dimensional environments for liver tissue engineering [38,51,52]. A gelatin cryogel enhanced differentiation of adipose-derived MSCs toward a hepatocyte-like phenotype, with increased hepatic marker expression and functional measures including urea production [38]. Incorporation of laminin into gelatin cryogels increased the proportion of differentiated cells compared with gelatin or laminin conditions alone and was associated with hepatic gene expression and functional maturation, including urea synthesis and glycogen storage [37]. These findings support the concept that combining complementary ECM signals may produce a more instructive microenvironment than a single ECM component.

Heparin represents another useful natural component because of its ability to interact with a broad range of growth factors and extracellular signaling molecules. In tissue-engineering systems, heparin can contribute to growth-factor retention and controlled presentation in addition to providing biochemical functionality. A micropatterned heparin-based hydrogel enhanced hepatic differentiation of human adipose-derived MSCs, including hepatic gene and protein expression and functional measures such as glycogen storage, albumin synthesis, and urea production [39]. A heparin/collagen scaffold similarly supported WJ-MSC differentiation, with albumin and cytokeratin 19 expression together with indocyanine green clearance [41]. These studies suggest that heparin-containing materials may act as biochemical reservoirs that complement soluble hepatogenic factors.

Alginate is a polysaccharide derived from brown algae and is frequently used for three-dimensional cell encapsulation because of its mild gelation conditions and tunable physical properties. Its relatively low intrinsic cell adhesiveness can be advantageous for controlling cell–material interactions but often motivates combination with ECM proteins or other biomolecules [53]. BM-MSCs cultured in calcium-crosslinked alginate scaffolds acquired hepatic characteristics, including expression of AFP, ALB, connexin 32, CYP7A1, and CK18, together with albumin and urea production and glycogen storage [32]. Three-dimensional alginate culture of UC-MSCs in conjunction with sequential hepatogenic induction also enhanced albumin and urea production [40]. Together, these studies illustrate how a relatively simple three-dimensional matrix can influence cell aggregation and exposure to differentiation signals.

Decellularized tissue-derived matrices provide a particularly biomimetic class of natural biomaterials. Decellularization removes cellular components while retaining aspects of the original ECM architecture and associated biochemical signals [54,55]. Decellularized liver matrix therefore offers an attractive strategy for recreating the organ-specific microenvironment. Liver-derived decellularized matrix promoted hepatic differentiation of adipose-derived MSCs more effectively than several coating matrices, including collagen, fibronectin, and Matrigel, when assessed by hepatic gene expression, protein production, and functional outcomes [36]. An acellular whole-liver scaffold supported more efficient generation of functional hepatocyte-like cells from BM-MSCs than conventional two-dimensional culture; the engineered tissue also showed therapeutic benefit in a mouse model of lethal liver failure [35].

Decellularized spleen-derived scaffolds represent an alternative tissue-derived platform. Decellularized splenic scaffolds retained native ECM and vascular microarchitecture and supported attachment, infiltration, and survival of BM-MSCs, providing a potential three-dimensional platform for liver tissue engineering [33]. Although the scaffold was not necessarily equivalent to a liver-specific matrix, the study highlights the potential of preserved organ architecture to support cell engraftment and tissue organization.

### 5.2. Synthetic Biomaterials

Synthetic biomaterials offer a complementary strategy because their physicochemical properties can be controlled more reproducibly than those of many naturally derived materials. Degradation rate, mechanical properties, porosity, swelling, and scaffold architecture can be tuned during fabrication [56]. Their principal limitations include relatively poor intrinsic cell adhesion and the possibility that degradation products or processing residues may adversely affect cell behavior [57]. Functionalization with ECM proteins or other bioactive molecules can address some of these limitations.

Poly(lactic-co-glycolic acid) (PLGA) is a biodegradable synthetic polymer widely investigated for tissue engineering because its composition and degradation can be adjusted. PLGA is particularly useful when combined with natural ECM components. As noted above, collagen-coated PLGA three-dimensional scaffolds improved hepatic metabolic function of BM-MSC-derived hepatocyte-like cells, including albumin secretion, urea synthesis, ammonia elimination, and diazepam clearance, compared with controls [34]. The study demonstrates the complementary roles of a synthetic structural framework and a natural biochemical interface.

Poly(L-lactic acid)-co-poly(ε-caprolactone) (PLACL) provides another synthetic platform with properties suitable for fibrous scaffold fabrication. Human MSCs cultured on PLACL/collagen nanofibrous scaffolds exhibited hepatosphere formation, hepatic marker expression, and albumin release; among the tested nanofibrous systems, the PLACL/collagen scaffold produced the strongest hepatogenic response [58]. The combination of nanofibrous architecture and collagen therefore provides an example of how synthetic topography and natural biochemical signals can be integrated to support hepatic differentiation.

Polyethylene glycol (PEG)-based materials offer highly tunable hydrogel chemistry and can be functionalized with ECM proteins. A PEG-based inverted colloidal crystal hydrogel functionalized with collagen I and fibronectin, in combination with Huh-7.5 cell coculture, enhanced hepatic gene expression, protein production, morphology, and metabolic function of human adipose-derived MSCs compared with less biomimetic conditions [29]. This study further emphasizes that synthetic materials can be engineered to become biologically instructive through controlled incorporation of ECM and cellular cues.

Taken together, an ideal biomaterial for MSC hepatic differentiation should provide more than structural support. It should be cytocompatible and reproducible; support cell adhesion, cell–cell interactions, and appropriate three-dimensional organization; permit adequate nutrient and oxygen transport; present hepatogenic biochemical cues; and provide mechanical properties that favor hepatic specification and maturation. For translational applications, the material should also be scalable, manufacturable, and compatible with clinically relevant culture conditions. Importantly, biomaterial performance should ultimately be evaluated by improvements in hepatic function and maturation rather than by increased expression of hepatic lineage markers alone.

## 6. Biomaterial Properties That Influence Hepatic Differentiation

The studies summarized above suggest that biomaterial effects cannot be attributed solely to material identity. Several interconnected properties, including three-dimensionality, ECM composition, growth-factor presentation, porosity, cell density, and mechanical environment, may determine how MSCs respond to a scaffold.

Three-dimensional organization is particularly important. Hepatocytes naturally exist within a highly organized multicellular architecture in which cell–cell contacts, ECM interactions, nutrient gradients, and vascular interfaces contribute to function. Three-dimensional aggregation can promote hepatosphere formation and improve cell survival and function, while recent work with MSC spheroids further demonstrates that three-dimensional organization can enhance hepatic differentiation and facilitate integration with vascularized tissue-engineering strategies [14,59]. Alginate scaffolds, cryogels, nanofibrous materials, and decellularized organs each provide different ways of generating such three-dimensional organization [32,33,35,37,38,40,58,60,61].

ECM composition provides a second layer of control. Collagen, laminin, fibronectin, and heparin can provide distinct cell-adhesion and growth-factor-binding signals, whereas decellularized liver ECM provides a more complex mixture of tissue-specific components [11,12,13,36,37,41]. These biochemical cues may cooperate with soluble factors to influence cell adhesion, cytoskeletal organization, survival, and differentiation. However, the relative contribution of individual ECM components remains incompletely defined, and many published studies use complex materials in which multiple variables change simultaneously.

Mechanical properties and microarchitecture represent additional variables that deserve greater attention. Synthetic materials permit systematic control of stiffness, porosity, degradation, topography, and viscoelasticity, whereas natural matrices may more closely reproduce biochemical cues [56,57,62,63]. Recent work using tunable biomaterial niches showed that matrix stiffness and stress relaxation can regulate MSC hepatic differentiation through mechanotransduction involving YAP, providing direct evidence that the physical state of a scaffold can influence hepatic fate [64]. Future studies should therefore move beyond simple comparisons of “natural” versus “synthetic” materials and instead determine which specific material properties drive hepatic specification and maturation. Such work could help identify optimal combinations of biochemical and biophysical cues rather than relying on empirical scaffold selection.

Biomaterial cues may also intersect with epigenetic regulation of hepatic fate. Histone acetylation is particularly relevant because chromatin accessibility can influence the activation of hepatogenic transcriptional programs. In three-dimensional collagen-based systems, histone deacetylase inhibition with valproic acid or sodium butyrate has been used to enhance hepatic differentiation of MSCs [65,66]. These findings support the concept that biomaterial-derived signals and epigenetic regulation may act cooperatively during hepatic differentiation. However, the interactions among material properties, mechanotransduction, and epigenetic state remain insufficiently characterized and represent an important area for future investigation. Key biomaterial-based studies investigating MSC hepatic differentiation and liver tissue engineering are summarized in Table 1.

## 7. Assessing Hepatic Differentiation: Phenotype Versus Function

A critical consideration in evaluating MSC hepatic differentiation is the distinction between the acquisition of a hepatic phenotype and the development of mature hepatocyte function. Hepatic differentiation studies commonly assess the expression of lineage-associated markers, including alpha-fetoprotein (AFP), albumin (ALB), cytokeratin 18 (CK18), cytokeratin 19 (CK19), hepatocyte nuclear factor 4 alpha (HNF4α), and cytochrome P450 (CYP) enzymes [42,43,44,45,46,47,48]. Although these markers provide important evidence of hepatic lineage commitment, expression of one or several hepatic markers alone does not necessarily demonstrate the generation of functionally mature hepatocytes.

Several of the biomaterial-based studies discussed in this review incorporated functional assays in addition to marker analysis. For example, BM-MSCs cultured in alginate scaffolds expressed hepatic markers and demonstrated albumin and urea production, CK18 expression, and glycogen storage [32]. Similarly, human MSCs cultured on PLACL/collagen nanofibrous scaffolds exhibited hepatic marker expression and albumin secretion, supporting the acquisition of hepatic characteristics in a three-dimensional biomaterial environment [58]. ECM-functionalized PEG-based hydrogels have also been associated with enhanced hepatic gene expression, protein production, and metabolic function in adipose-derived MSCs [29]. Gelatin/laminin cryogels promoted hepatic differentiation together with functional outcomes including urea synthesis and glycogen storage [37], while heparin/collagen scaffolds supported hepatic marker expression and indocyanine green clearance in Wharton’s jelly-derived MSCs [41]. Similar functional endpoints—including albumin secretion, urea production, glycogen storage, and metabolic activity—have been reported in collagen-coated PLGA scaffolds, gelatin cryogels, heparin-based hydrogels, and decellularized liver scaffolds [34,35,38,39].

These findings highlight the importance of using multiple complementary endpoints to characterize MSC-derived hepatocyte-like cells. Phenotypic assessment can include hepatic transcription factors and structural or secretory proteins such as HNF4α, ALB, CK18, CK19, alpha-1-antitrypsin, and other liver-associated markers. However, functional characterization should ideally include assays that reflect major hepatocyte activities, including albumin secretion, urea synthesis, glycogen storage, ammonia metabolism, and uptake or transport of hepatic substrates. Indocyanine green uptake or clearance can provide evidence of hepatic uptake capacity, whereas cytochrome P450 expression and, preferably, enzyme activity provide information regarding xenobiotic metabolism. Functional characterization of MSC-derived hepatocyte-like cells has specifically included inducible CYP-dependent activity, supporting the importance of metabolic assays beyond marker expression [22]. In a direct characterization study of hepatocyte-like cells derived from human MSCs, assessment of albumin secretion, hepatic gene expression, glycogen storage, indocyanine green uptake, and hepatic polarization demonstrated that functional characteristics may differ substantially from those of more mature hepatocyte populations [23].

An additional consideration is cellular maturation. Hepatocyte-like cells generated from stem or stromal cells may acquire several hepatic characteristics while retaining features associated with immature or fetal hepatic cells. Therefore, the term “hepatocyte-like cell” should not automatically be interpreted as equivalent to a mature adult hepatocyte. Recent recommendations for characterization of in vitro-generated hepatocytes emphasize the importance of assessing multiple dimensions of hepatic identity and function and, where possible, benchmarking the generated cells against primary human hepatocytes as a reference standard [24]. For biomaterial-based MSC differentiation studies, this distinction is particularly important because a scaffold may increase hepatic marker expression without necessarily producing a proportional improvement in mature metabolic function.

Accordingly, future studies should adopt a multidimensional characterization strategy that combines hepatic lineage markers, quantitative functional assays, and indicators of maturation. As a practical minimum evidence set, MSC-derived hepatocyte-like cells should demonstrate (i) coherent hepatic identity using multiple markers, including a hepatic transcriptional regulator such as HNF4α together with mature hepatic proteins such as ALB, alpha-1-antitrypsin, or CK18; (ii) at least two quantitative hepatic functions representing distinct functional domains, including a synthetic or metabolic function such as albumin secretion or urea synthesis together with an additional function such as glycogen storage, ammonia metabolism, or hepatic substrate uptake/transport; and (iii) evidence of xenobiotic-metabolizing competence, preferably through CYP enzyme activity rather than CYP expression alone. For studies claiming advanced hepatic maturation, additional assessment of hepatic transporter activity, cellular polarity, bile canalicular organization, and long-term functional stability would provide stronger evidence. Where feasible, these parameters should be benchmarked against primary human hepatocytes or another well-characterized mature hepatic control. This proposed minimum set is intended as a practical framework for evaluating biomaterial-mediated hepatic differentiation rather than as a universal regulatory potency assay.

## 8. Future Perspectives

Biomaterial-based MSC hepatic differentiation has progressed from simple scaffold-supported culture toward increasingly complex three-dimensional microenvironments. Nevertheless, several challenges must be addressed before these systems can be translated into robust therapeutic platforms. First, the generation of mature hepatocyte-like cells remains inconsistent. Many studies demonstrate hepatic marker expression and selected functions, but complete acquisition of mature metabolic and polarization profiles remains difficult.

Second, biomaterial systems need greater standardization. MSC source, donor characteristics, passage number, differentiation media, scaffold composition, crosslinking conditions, culture duration, and functional assays differ widely among studies. This heterogeneity makes it difficult to identify which biomaterial features are genuinely responsible for improved hepatic outcomes. Standardized reporting and head-to-head comparisons of material properties will be important for progress.

Third, future scaffolds should increasingly be designed as instructive rather than passive materials. A next-generation biomaterial could integrate ECM ligands, controlled growth-factor presentation, tunable mechanical properties, and three-dimensional architecture within a single system. Dynamic or spatially patterned materials may further reproduce the changing biochemical and physical environment encountered during hepatic development and regeneration. Recent evidence that matrix stiffness and stress relaxation can regulate MSC hepatic differentiation through YAP-mediated mechanotransduction further suggests that these physical properties should be treated as deliberate biomaterial design variables [64].

Epigenetic regulation should also be considered within this instructive-material framework. Histone deacetylase inhibition with valproic acid or sodium butyrate has enhanced MSC hepatic differentiation in three-dimensional collagen-based systems [65,66], providing proof-of-concept that biomaterial environments and epigenetic modulation can be combined. Future studies should determine whether specific biomaterial properties themselves induce reproducible epigenetic changes that promote hepatic specification and maturation.

Fourth, the therapeutic effects of MSCs should not be evaluated solely according to their capacity to become hepatocytes. MSC-derived soluble factors and extracellular vesicles can influence inflammation, fibrosis, angiogenesis, and endogenous tissue repair. Biomaterials that improve MSC retention and regulate the secretome may therefore promote liver regeneration even when direct differentiation is incomplete. Recent work using 3D-printed vascularized artificial liver lobules incorporating MSC spheroids and spheroid-derived extracellular vesicles illustrates how biomaterial architecture, multicellular organization, and MSC paracrine activity can be integrated within a regenerative platform [67]. Integrating differentiation and paracrine mechanisms represents an important direction for future tissue-engineering studies.

Finally, the native liver is highly organized, vascularized, and spatially heterogeneous. Hepatocytes interact with sinusoidal endothelial cells, hepatic stellate cells, immune cells, and a specialized ECM. The generation of vascularized liver organ buds has demonstrated the importance and feasibility of incorporating multicellular organization and vascular components into engineered hepatic tissues [68]. Reproducing this organization will likely require composite or multicellular biomaterial systems rather than a single homogeneous scaffold. The long-term objective should therefore be the development of scalable, reproducible, and clinically compatible biomaterials that coordinate cellular, biochemical, mechanical, epigenetic, and vascular cues to generate functional hepatic tissue.

## 9. Conclusions

MSCs remain an attractive platform for liver regeneration because of their accessibility, expansion capacity, immunomodulatory activity, and regenerative secretome. However, efficient generation of mature hepatocyte-like cells remains a major challenge, and soluble differentiation factors alone do not fully reproduce the complex hepatic microenvironment. The studies reviewed here demonstrate that natural biomaterials, including collagen, gelatin, laminin-containing matrices, heparin, alginate, and decellularized tissue ECM, can provide biochemical and three-dimensional cues that complement hepatogenic media. Synthetic materials such as PLGA, PLACL, and PEG offer additional control over architecture and physicochemical properties and can be enhanced through ECM functionalization. Overall, the field is moving from using biomaterials as passive scaffolds to using them as instructive microenvironments. Future progress will depend on defining the specific biochemical and biophysical properties that promote durable hepatic function, integrating differentiation with MSC paracrine activity, and developing biomaterial systems that more faithfully reproduce the spatial, vascular, and multicellular organization of native liver tissue.

## Figures and Tables

**Table 1:** Biomaterials used in MSC hepatic differentiation and liver tissue engineering.

Biomaterial	MSC/Cell Source	Differentiation Conditions	Principal Outcome	Ref.
Decellularized liver matrix; collagen; fibronectin; Matrigel	AD-MSCs	bFGF, HGF, OSM, dexamethasone, ITS	Liver-derived decellularized matrix produced higher AFP, ALB, urea, and hepatic functional responses than comparator coating matrices.	[36]
Collagen-coated PLGA 3D scaffold	BM-MSCs	HGF, dexamethasone, OSM, ITS	Enhanced hepatic metabolic functions, including albumin secretion, urea synthesis, ammonia elimination, and diazepam clearance.	[34]
Acellular whole-liver scaffold	BM-MSCs	HGF, bFGF, nicotinamide, OSM, dexamethasone, ITS	Enhanced generation of functional hepatocyte-like cells compared with 2D culture; engineered tissue showed therapeutic benefit in a mouse liver-failure model.	[35]
Calcium-crosslinked alginate scaffold	BM-MSCs	IMDM, FBS, ITS, dexamethasone, nicotinamide, ascorbic acid-2-phosphate, EGF, HGF, FGF-4	Supported 3D organization and hepatic differentiation, with hepatic markers, albumin/urea production, CK18 expression, and glycogen storage.	[32]
PEG-based inverted colloidal crystal hydrogel functionalized with collagen I/fibronectin	AD-MSCs	HGF, FGF-1, FGF-4, hydrocortisone, nicotinamide, ascorbic acid, ITS, dexamethasone, OSM	ECM-functionalized 3D culture and Huh-7.5 coculture enhanced hepatic gene expression, protein production, morphology, and metabolic function.	[29]
Calcium-crosslinked alginate hydrogel beads	UC-MSCs	FGF4, HGF, dexamethasone, glucagon, ITS, OSM, TSA	Three-dimensional alginate culture combined with sequential induction increased albumin and urea production.	[40]
Micropatterned heparin-based PEG-DA hydrogel	AD-MSCs	OSM, nicotinamide, dexamethasone, ITS, HGF, EGF, bFGF	Enhanced hepatic gene/protein expression and metabolic functions, including glycogen storage, albumin, and urea production.	[39]
Decellularized splenic ECM scaffold	BM-MSCs	DMEM/F12 + 10% FBS	Supported MSC attachment, infiltration, viability, and engraftment within a native-like 3D ECM and vascular microarchitecture.	[33]
Laminin-containing gelatin cryogel	AD-MSCs	DMEM, FBS, dexamethasone, HGF, OSM	Enhanced hepatic marker expression and liver-specific functions, including urea synthesis and glycogen storage.	[37]
3D gelatin cryogel	AD-MSCs	HGF, dexamethasone, OSM, basal growth medium	Promoted hepatocyte-like morphology, ALB/AFP/CK18/CK19 expression, urea production, and glycogen storage.	[38]
PLACL/collagen nanofibrous scaffold	hMSCs	HGF, bFGF, nicotinamide, OSM, dexamethasone, ITS	Supported hepatosphere formation and hepatic differentiation; the PLACL/collagen formulation produced the strongest hepatogenic response among tested scaffolds.	[58]
Heparin–collagen type I scaffold	WJ-MSCs	HGF, IGF-1, OSM; basal DMEM + FBS and supplements	Enhanced hepatic marker expression and metabolic activity, including albumin, CK19, and indocyanine green clearance.	[41]
dECM/oxidized-dextran hydrogel with tunable mechanics	AD-MSCs	Hepatogenic induction within hydrogels with varied stiffness and stress relaxation	Liver-like low stiffness and faster stress relaxation promoted hepatic differentiation through YAP-associated mechanotransduction and improved therapeutic effects in experimental acute liver failure.	[64]
3D-printed vascularized artificial liver lobules with MSC spheroids/SpEVs	MSCs with patterned endothelial cells	3D spheroid culture, endothelial coculture, and spheroid-derived EV integration	MSC spheroids showed enhanced hepatic differentiation; integration with vascular patterning and SpEVs improved pro-regenerative functions and therapeutic efficacy in liver failure.	[67]

## Data Availability

No new data were created or analyzed in this study. Data sharing is not applicable to this article.

## Abbreviations

ALB—Albumin; AFP—Alpha-fetoprotein; AAT—Alpha-1-antitrypsin; AD-MSCs—Adipose-derived mesenchymal stromal cells; BM-MSCs—Bone marrow-derived mesenchymal stromal cells; ECM—Extracellular matrix; EGF—Epidermal growth factor; FGF—Fibroblast growth factor; HGF—Hepatocyte growth factor; IGF-1—Insulin-like growth factor-1; IMDM—Iscove’s modified Dulbecco’s medium; ITS—Insulin-transferrin-selenium; OSM—Oncostatin M; PEG—Polyethylene glycol; PEG-DA—Polyethylene glycol diacrylate; PLACL—Poly(L-lactic acid)-co-poly(ε-caprolactone); PLGA—Poly(lactic-co-glycolic acid); TSA—Trichostatin A; UC-MSCs—Umbilical cord-derived mesenchymal stromal cells; WJ-MSCs—Wharton’s jelly-derived mesenchymal stromal cells.
